# Evolution in Dentistry

**Published:** 1890-09

**Authors:** J. A. Robinson


### Evolution in Dentistry.

BY J. A. ROBINSON, D.D.S.

Evolution is improved continuity of life and action derived from a previous fact; it is the law of becoming; it does not go back to the origin of causes, but takes things as they exist and carries them forward and upward.

The origin of life and society is too distant and too obscure; we must take life and society as we find it in history and learn what we can from the history of those who have gone before, and this, with the solicitation of a friend, is what prompted the writing of Evolution in Dentistry. It is the recollections of some thing that has taken place, perhaps before the present generation was born; it is the next in degree according to a fixed law of progress. Natural selection, according to Mr. Darwin, is intent on preserving the best individuals, and so society is intent on preserving the best laws, and mechanics in preserving the best principles. Our civilization seems to be like the clay in the hands of the potter who makes the vessel and the clay is the antecedent of civilization that has gone before us. We are acted upon. Government, laws and arts seem to be forced upon us. Events and ideas are a necessary sequence that follow an irresistible law and that is evolution.

Dentistry is largely the product of the age in which we live; it corresponds exactly to our culture and civilization. As material wealth is the base of all refinement and culture and as immense fortunes are made and confined mostly to large cities, so dentistry flourishes, as a rule, in proportion to the number of inhabitants and wealth and culture of the people. Sixty years ago there were only half a dozen dentists in Boston, and only half a hundred in all New England, but now there is a dentist in every town with a population of twenty-five hundred persons.

It is the weaving of the web in the loom of time to raise humanity to a higher level. George Washington was the only man of his time, in history of whom we have any account, who wore a set of artificial teeth. These were made by John Greenwood, of New York—he was the foremost man in America and was the product of the Revolution, as Mr. Lincoln and General Grant were the foremost men in our late civil war, and were the product of the rebellion. Corydon Palmer had been fitted for fine operations on the teeth and the manufacture of dental instruments while he worked in a watch shop; his big hand was obedient to his keen perceptive faculties and thoughtful mind and it made him one of the foremost operators, as soon as his mind turned to the subject of dentistry.

Dr. W. H. Atkinson has always stood in the same relation to dentistry that Patrick Henry stood to the patriots in our old revolutionary war; he was an inspirer of the minds of other men, he could see the end and stimulate a whole convention to higher aims and loftier achievements Dr. C. R. Butler was a pupil of Dr. Atkinson's and in him were combined the enthusiasm of Atkinson with the fine manipulative qualities of Corydon Palmer. As the orator is made when the logic and the heart are united in the same individual so the perfect dentist is reached when fine mechanical qualities are combined in the heart and concentrated in the same person.

When the *News Letter* was merged into the *Cosmos*, Dr. Butler was the umpire to decide what instruments should be reproduced by the new White Manufacturing Establishment and many new forms were submitted to his judgment for real practical utility besides those he designed himself known as "Butler's Pluggers," that have rarely ever been surpassed for practical use in filling teeth.

One pleasant morning while on a visit to my son, in Cleveland, I called to see Dr Butler. He answered the call of the door bell in person, took me by both hands for a shake and said "Come in and stand by my chair and see me fill a tooth for which I get twenty dollars, *for you are the only old man who ever kept up.*" I considered it quite a compliment, and stayed with him half a day and saw one of the best fillings I had ever seen.

The average of Dr. Butler's dentistry like Dr. McKellops', of St. Louis, is *first class*, because they are artists. It is not what we do sometimes on extra occasions that makes a first class operator, but what we do always, what we do because we can not do otherwise than make the best.

I have used the names of these persons to illustrate a point in evolution in dentistry, to show the point of separation between the really great and mediocrity. We all belong to humanity and there is no missing link; we have all one common purpose viz.: getting a living; we may all possess the same moral, or the same social, but not the same *artistic qualities*, and it requires the trinity of forces to become a dentist. Neither have I used these names as being better operators than all other men but because I happened to know more of them, for the country is full of good dentists; but I spent a week with Dr. McKellops a few years after the war and was particularly struck with the excellent quality of his operations.

The Butler set of pluggers were the first I ever knew that claimed to be carried forward in any methodical manner. I do not recollect the exact number that comprised his set, but No. 16 is one of the most useful that has been given to the public, so there were as many, or more than that number. Then came the Varney, and then the Watling, and hence about as many names as there are letters in the alphabet.

The corkscrew plugger is as old as dentistry only it was brought to a sharp point like a cow's horn, afterward it was squared on the end and serrated and makes a part of a set in most cases. I wrote up pluggers a dozen years ago and recommended a stout knifeblade plugger with three points and the serrates running up the sides for carrying the gold to the margin of the cavities and finishing with an oval-faced mallet burnisher for all contour fillings to prevent breaking the edges of the enamel. All pluggers were contrived to subserve some useful purpose like the rudimentary organs in the animal economy, but having served that purpose as we make progress in methods they are laid aside like the turnkey in extracting teeth. Our methods of manipulation change and they are no longer necessary.

Like the origin of forms in animal life they are a necessity, but as habits and civilization change they come into disuse and change and almost all that follow are derivatives, and this is evolution.

At the last State Dental Society meeting in this city the members had a *tilt* with Dr. Loyd L. Davis for violating the code of ethics by advertising and suspended him for one year. Dr. Davis did a very indiscreet thing in the wording of his circular if he expected to escape censure. When called upon to say what he had to say in his defence his words were so guarded that what he said was about as much a defence of his course as an apology, and the members were not satisfied. He would not say he was *sorry* and it was evident that he was not sorry at all; he has risen so far above the views of the majority that he would not tell a lie to escape the penalty. In that thing he is to be commended, and it was unkind in the majority to ask him to tell a palpable falsehood before a hundred of his brethren. Moral truth is a divine power in the nature of man. It may be born with us, or is a creature of education. Conscience is above all creeds, or liturgies, or codes of ethics. The travail of a man's soul begins in trying to reform the abuses of the age in which he lives. The dignity of any body of men should never be raised to such a degree as to deprive the humblest member of his natural rights. The victims of codes that have outlived their usefulness are bound in mental and sentimental slavery.

The law of progress in society and the ruling power in the universe is love; it is always trying to reproduce its kind, and to save its life after its reproduction. This is admirably illustrated in the professions. Take our own for instance. What prompts the dentist to excel in his profession? It is because he sees in his patient a poor set of teeth, a swollen face and disfigured countenance, and a pain that he can remove and make the poor suffering unfortunate better and happier. Of course he expects compensation for his services, for that is the next law of life, but if he is fit for his calling the ruling power of his life is love, or he is not fit to be employed. It is the same with the physician and then the lawyer (if he is good for any thing) must have the good of society as his strongest motive to action and not his love for fees. The minister also is always striving to make his flock better that they may be happier here and hereafter. It is only when we are drawn away by the love of self or mistaken views of society that we act contrary to this. This desire to make things better is what induced the writer to line with metal the rubber dental plate with *fibrous metallic lining.* As the fibrous lining did not last more than five or six years Dr. Albert Robinson, invented and introduced a gold lining that is a perfect success and makes a rubber plate lined with a gold lining the perfection of an artificial denture.

The next step in progress over the rubber plate was that made by Dr. S. L. Edwards, of Des Moines, Iowa. It is made by covering the cast with Japanese tea lead and burnishing it into all the inequalities, while the shellac varnish is drying. That gives it a smooth hard surface after the teeth are articulated. Now adjust another plate over the entire lingual surface just above the wax line and burnish round the teeth to prevent the plaster from working between the lead plates while flasking. When set remove the wax with clean hot water. When you close the flask insert an iron wedge by each bolt the thickness you desire the plate and you will have a smooth plate on both surfaces the thickness of the wedge you place between the two points of the flask.

There is quite a difference of opinion among dentists as to the cause of the softening and absorption of the alveolar ridge, but a gold-lined plate will prevent or cure any and all inflamed mouths, and that is evolution in rubber work. The old gold plate never inflamed the mouth. I know of some that have been worn with perfect comfort more than forty years that are in a good state of preservation to day, and the alveolar ridge is almost as perfect as when the plate was first worn.

